# A macroscopic approach for stress-driven anisotropic growth in bioengineered soft tissues

**DOI:** 10.1007/s10237-021-01554-1

**Published:** 2022-01-19

**Authors:** L. Lamm, H. Holthusen, T. Brepols, S. Jockenhövel, S. Reese

**Affiliations:** 1 Institute of Applied Mechanics, RWTH Aachen University, Mies-van-der-Rohe-Str. 1, 52074 Aachen, Germany; 2 Biohybrid and Medical Textiles, Institute of Applied Medical Engineering, RWTH Aachen University, Pauwelsstr. 20, 52074 Aachen, Germany

**Keywords:** Anisotropic growth, Growth potential, Engineered tissue, Finite strain

## Abstract

The simulation of growth processes within soft biological tissues is of utmost importance for many applications in the medical sector. Within this contribution, we propose a new macroscopic approach for modelling stress-driven volumetric growth occurring in soft tissues. Instead of using the standard approach of a-priori defining the structure of the growth tensor, we postulate the existence of a general growth potential. Such a potential describes all eligible homeostatic stress states that can ultimately be reached as a result of the growth process. Making use of well-established methods from visco-plasticity, the evolution of the growth-related right Cauchy–Green tensor is subsequently defined as a time-dependent associative evolution law with respect to the introduced potential. This approach naturally leads to a formulation that is able to cover both, isotropic and anisotropic growth-related changes in geometry. It furthermore allows the model to flexibly adapt to changing boundary and loading conditions. Besides the theoretical development, we also describe the algorithmic implementation and furthermore compare the newly derived model with a standard formulation of isotropic growth.

## Introduction

The production and use of artificially grown biological tissue has become an important research topic in the medical context over the last two decades. Great progress has been made in implant research in particular, with the cultivation of biohybrid heart valves being just one example among many (Fioretta et al. 2019). Designing and constructing highly complex medical implants is a big challenge due to the biomechanical properties of the underlying cultivated tissue. Early works in the field of biomechanics have already pointed out that biological tissues adapt dynamically to the environment they are exposed to (see e.g. Fung 1995 and references therein). The goal of this process is to reach a homeostatic state in which e.g. a certain critical stress state is neither exceeded nor fallen below. From a physiological point of view this process, which we will call growth in the following, is mainly driven by a change in mass and internal structure of the given biological material. It is important to notice that since the model presented in this paper is a purely phenomenological approach, we disregard the micromechanical effects of remodelling in the following and concentrate exclusively on the description of volumetric growth. Subsequently, the growth process leads to a change in the mechanical behaviour, which usually has a large influence on the performance of the given implant. In contrast to native tissue, these adaptive effects are particularly pronounced during the cultivation period of bioengineered tissues and must therefore be taken into account from the beginning of the design process. Within this context, computational modelling contributes to a deeper understanding and prediction of such adaptation processes. An important aspect of modelling the mechanics of growth is the description of geometry changes which are due to contraction and expansion of the material, respectively. Starting from the works of Skalak (1981), Skalak et al. (1982) and Rodriguez et al. (1994), many models have been developed over the last decades in order to describe such finite volumetric growth effects. Although being already successfully applied, e.g. in the modelling of finite plasticity (see e.g. Eckart 1948; Kröner 1959; Lee 1969), it was the contribution of Rodriguez et al. (1994) which first adapted the multiplicative split of the deformation gradient to describe the inelastic nature of finite growth processes. For a detailed overview on the various modelling strategies, the interested reader is referred to the comprehensive overviews given e.g. by Goriely (2017) and Ambrosi et al. (2019). Most of the approaches based on the conceptually simple and computationally efficient framework by Rodriguez et al. (1994) can be roughly divided into two different groups, isotropic (e.g. Lubarda and Hoger 2002; Himpel et al. 2005) and anisotropic growth models (e.g. Menzel 2005; Göktepe et al. 2010; Soleimani et al. 2020). It is important to outline that the terms *isotropic* and *anisotropic* which are used here in the context of describing the volumetric growth response must not be confused with the similar terminology of the underlying basic continuum mechanical models, where *anisotropy* is often used to denote an initially preferred direction within the material. In case of isotropic growth, the growth-related part of the deformation gradient tensor is often assumed to be proportional to the identity tensor (e.g. Lubarda and Hoger 2002), which yields a uniform expansion of a referential volume element. On the other hand, the term anisotropic growth describes a geometry change of a given volume element that is not uniform in all three spatial dimensions but rather has a distinct growth direction (e.g. Göktepe et al. 2010). Despite its widespread use, the approach of isotropic growth modelling has strong limitations with regard to describing the mechanical behaviour of complex structures. Recently, (Braeu et al. 2017, 2019) pointed out that in the context of relevant applications, anisotropic growth behaviour is more the standard case than an isotropic response. Classically, this intrinsically anisotropic growth behaviour is modelled using heuristic assumptions on the definition of preferred growth directions. This, unfortunately, yields the need to a-priori prescribe a certain structure of the growth-related deformation gradient. While this approach might be feasible for relatively simple problems such as fibre elongation and contraction, it is not well applicable for more complex applications. In order to cure the need for describing the structure of the growth-related deformation gradient a-priori, more recent works (e.g. Zahn and Balzani 2017) have developed formulations in which the growth-related deformation gradient tensor is constructed with respect to the eigenvectors corresponding to the principal stress state. Nevertheless, defining general and flexible formulations that can adapt to various boundary value problems remains a challenging task and ongoing topic of research, as pointed out already by e.g. (Menzel 2005) or (Soleimani et al. 2020).

In addition to the phenomenologically motivated models described above, another class of models was established for describing growth processes. Originating from the theory of mixtures, (Humphrey and Rajagopal 2002), among others, developed the constrained mixture theory. Instead of assuming that the volume as a whole is deformed during the growth process, this modelling approach describes the change of volume in terms of a continuous deposition and removal of mass increments. Since this approach is computationally very expensive, (Cyron et al. 2016) and (Cyron and Humphrey 2017) developed a homogenized version of the constrained mixture model. This is achieved by using a temporal homogenization of the mass increments alongside with the same multiplicative split as described by Rodriguez et al. (1994). Although this approach overcomes the limitations of the classical constrained mixture theory in terms of computational costs, it still suffers from the need to a-priori define the structure of the growth tensor. Recent versions of this framework, as described e.g. in the work of Braeu et al. (2019), were able to modify this approach such that the growth tensor adapts automatically to the given boundary value problem.

As an alternative to the just mentioned promising approach, this contribution presents a different way on tackling the issue of predefined growth tensors. This novel and flexible framework for the description of stress-driven volumetric growth is able to cover both, isotropic and anisotropic growth behaviour, naturally. Section 2 covers the theoretical modelling ideas behind the proposed model. The numerical implementation of the derived material model is described in Sect. 3. Finally, numerical examples are given in Sect. 4.

## Continuum mechanical modelling of finite growth

Let us first introduce the well-established multiplicative split of the deformation gradient $\mathbf {F}$ into an elastic and a growth-related part (see e.g. Rodriguez et al. 1994), i.e.

1 $$\begin{aligned} \mathbf {F} = \mathbf {F}_{\rm e}\mathbf {F}_{\rm g}. \end{aligned}$$

Using this equation, the determinant of $\mathbf {F}$, abbreviated by $J:={\text {det}}\,\mathbf {F} = {\text {det}}\,\mathbf {F}_{\rm e} {\text {det}}\,\mathbf {F}_{\rm g}$, is also multiplicatively split into two parts. While the change of volume due to elastic deformations is described by $J_{\rm e} = {\text {det}}\,\mathbf {F}_{\rm e}$, the growth-related volume changes are represented by $J_{\rm g} = {\text {det}}\,\mathbf {F}_{\rm g}$. In analogy to the right Cauchy–Green tensor $\mathbf {C} = \mathbf {F}^{T}\mathbf {F}$ as well as the left Cauchy–Green tensor $\mathbf {B}=\mathbf {F}\mathbf {F}^{T}$, the elastic right Cauchy–Green tensor and the growth-related right and left Cauchy–Green tensor can be defined as

2 $$\begin{aligned} \begin{aligned} \mathbf {C}_{\rm e}&:=\mathbf {F}_{\rm e}^{T}\mathbf {F}_{\rm e} = \mathbf {F}_{\rm g}^{-T}\mathbf {C}\mathbf {F}_{\rm g}^{-1}\\ \mathbf {C}_{\rm g}&:=\mathbf {F}_{\rm g}^{T}\mathbf {F}_{\rm g}\\ \mathbf {B}_{\rm g}&:=\mathbf {F}_{\rm g}\mathbf {F}_{\rm g}^{T}. \end{aligned} \end{aligned}$$

Furthermore, the growth-related velocity gradient $\mathbf {L}_{\rm g}$ is introduced as

3 $$\begin{aligned} \mathbf {L}_{\rm g} = \dot{\mathbf {F}}_{\rm g}\mathbf {F}_{\rm g}^{-1}. \end{aligned}$$

### Balance relations

Growth processes within biological systems in general lead to a change of the systems mass as well as a change of its shape and volume, respectively. Within this contribution, the focus lies on the macroscopic description of changes in shape rather than a change of the systems mass. We therefore neglect the description of the balance of mass in terms of production or flux terms and assume that this balance relation is fulfilled implicitly. It is furthermore well established to assume that growth processes take place on a significantly larger time scale than mechanical deformations do. This standard argument is known as the *slow growth assumption* and yields a quasi-static setup of the well known balance of linear momentum

4 $$\begin{aligned} {\text {Div}}\,\left( \mathbf {F}\mathbf {S}\right) + \mathbf {b}_0 = \mathbf {0}. \end{aligned}$$

Here, $\mathbf {S}$ and $\mathbf {b}_0$ denote the second Piola–Kirchhoff stress tensor and the referential body force vector per reference volume, respectively. Following the idea of open system thermodynamics (see e.g. Kuhl and Steinmann 2003 and references therein), we describe the entropy production $\dot{\gamma }$ in terms of the Clausius–Duhem inequality

5 $$\begin{aligned} \dot{\gamma } = \mathbf {S} : \frac{1}{2}\dot{\mathbf {C}} - \dot{\psi } + \mathcal {S}_0 \ge 0, \end{aligned}$$

with the volume specific Helmholtz free energy density $\psi$ defined more precisely in the following section. The additional referential entropy contribution $\mathcal {S}_0$ is capturing both, entropy fluxes through the boundary as well as entropy sources within the system itself. It is important to notice that we do not explicitly compute this particular contribution but introduce it to allow e.g. for a decrease in entropy due to the growth process itself.

### Helmholtz free energy

We start from the general continuum mechanical framework laid down in Svendsen (2001). Within this context, the constitutive equations are described with respect to a given but otherwise arbitrary configuration of the material body in question. Similar to the approaches made by Bertram (1999) and Svendsen (2001) in the context of finite plasticity, we choose the elastic part of the Helmholtz free energy to be stated in terms of quantities defined within the so-called grown intermediate configuration.

When modelling finite volumetric growth, it is important to ensure that the growth process will ultimately reach a homeostatic state. This must be the case even under the absence of growth restricting boundary conditions, since, otherwise, the growth process would continue ad infinitum. A common approach to limit the growth response is to introduce a set of material parameters, which can be interpreted as the maximum possible growth induced stretches (see e.g. Lubarda and Hoger 2002). Such approaches may give computationally reasonable results, however, in the authors’ opinion, cannot be easily motivated by physical arguments. Within this contribution we much rather assume that an internal force must evolve during the growth process that consequently counteracts the deformation process and ultimately yields it to stop. Since (engineered) biological tissue consists of high amounts of bound water, it is reasonable to assume that a growth-related change in volume is always accompanied by a change in internal pressure. Such pressure accumulations are consequently counteracting the expansion and contraction process, respectively. This growth-related internal pressure can be described by including an additional dependency on either $\mathbf {C}_{\rm g}$ or $\mathbf {B}_{\rm g}$. Using the idea of interpreting $\mathbf {F}_{\rm g}$ as a so-called *material isomorphism* (see e.g. Noll 1958; Svendsen 2001), it follows that one has to choose $\mathbf {B}_{\rm g}$ in order to ensure that the kinematic quantities are located within the same configuration, i.e.

$$\begin{aligned} \psi := \tilde{\psi }\left( \mathbf {C}_{\rm e}, \mathbf {B}_{\rm g}\right) . \end{aligned}$$

**Fig. 1:**
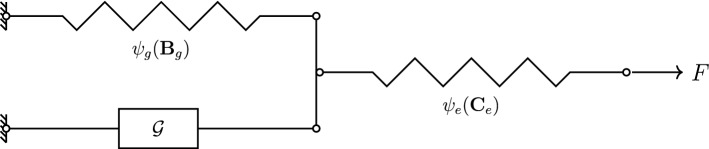
Rheological model corresponding to the given volumetric growth model. Growth is denoted by the element including the character $\mathcal {G}$

Note that this choice is strongly related to the general concept of structural tensors. In the present case, namely by choosing the structural tensor equal to $\mathbf {B}_{\rm g}$, the relation to linear kinematic hardening becomes obvious. This is worked out in the paper of Dettmer and Reese (2004), where linear kinematic hardening is a special case of the so-called Armstrong–Frederick type of kinematic hardening. In the following, we choose an additive format, i.e.

6 $$\begin{aligned} \tilde{\psi } := \psi _{\rm e}(\mathbf {C}_{\rm e}) + \psi _{\rm g}(\mathbf {B}_{\rm g}), \end{aligned}$$

for the Helmholtz free energy, which can be motivated easily by the rheological model shown in Fig. 1.

This model illustrates nicely that a growth-related expansion or contraction directly results in an accumulation of the growth-related energy $\psi _{\rm g}$ due to the loading of the associated spring element. Such an increase in growth-related energy clearly counteracts the growth deformation and ultimately leads to a decaying growth response. Please notice that the general idea of an energy contribution counteracting the growth process can also be found e.g. in Braeu et al. (2019). In the above publication, however, this is achieved by a change in elastic energy, which is enforced by an additional term considering the change in volume directly. The elastically stored energy $\psi _{\rm e}$ is represented within this rheological model by the second spring element. It is obvious that this particular spring is influenced by both, growth-related and purely elastic deformations.

### Thermodynamic considerations

To derive the constitutive equations representing finite volumetric growth, we next consider the isothermal Clausius–Duhem inequality as given in Eq. (5). Inserting the Helmholtz free energy (Eq. 6) and differentiating with respect to time yields

7 $$\begin{aligned}&\mathbf {S} : \frac{1}{2}\dot{\mathbf {C}} - \left( \frac{\partial \psi _{\rm e}}{\partial \mathbf {C}_{\rm e}} : \dot{\mathbf {C}}_{\rm e} + \frac{\partial \psi _{\rm g}}{\partial \mathbf {B}_{\rm g}}:\dot{\mathbf {B}}_{\rm g}\right) + \mathcal {S}_0 \ge 0. \end{aligned}$$

By using the product rule as well as utilizing the identities $\dot{\overline{\mathbf {F}_{\rm g}^{-T}}} = -\mathbf {F}_{\rm g}^{-T}\dot{\overline{\mathbf {F}_{\rm g}^{T}}}\mathbf {F}_{\rm g}^{-T}$ and $\dot{\overline{\mathbf {F}_{\rm g}^{-1}}} = -\mathbf {F}_{\rm g}^{-1}\dot{\mathbf {F}}_{\rm g}\mathbf {F}_{\rm g}^{-1}$, the elastic deformation rate can be expressed as

8 $$\begin{aligned}\dot{\mathbf {C}}_{\rm e} = \mathbf {F}_{\rm g}^{-T}\dot{\mathbf {C}}\mathbf {F}_{\rm g}^{-1} - \mathbf {L}_{\rm g}^{T}\mathbf {C}_{\rm e} - \mathbf {C}_{\rm e}\mathbf {L}_{\rm g}. \end{aligned}$$

With the definition of the growth velocity gradient given in Eq. (3), the growth-related deformation rate can similarly be found as

9 $$\begin{aligned} \dot{\mathbf {B}}_{\rm g} = \mathbf {L}_{\rm g}\mathbf {B}_{\rm g} + \mathbf {B}_{\rm g}\mathbf {L}_{\rm g}^{T}. \end{aligned}$$

As shown in detail in “Appendix 1”, the thermodynamically consistent definition of the second Piola–Kirchhoff stress tensor can be derived by combining the equations above and making use of the standard procedure of Coleman and Noll (1963), such that

10 $$\begin{aligned} \mathbf {S} = 2\mathbf {F}_{\rm g}^{-1}\frac{\partial \psi }{\partial \mathbf {C}_{\rm e}}\mathbf {F}_{\rm g}^{-T}. \end{aligned}$$

With this definition at hand, the reduced version of the Clausius–Duhem inequality is given as ${ \dot{\gamma }_{\rm red} = \frac{\partial \psi _{\rm e}}{\partial \mathbf {C}_{\rm e}} : \left( \mathbf {L}_{\rm g}^{T}\mathbf {C}_{\rm e} + \mathbf {C}_{\rm e}\mathbf {L}_{\rm g}\right) - \frac{\partial \psi _{\rm g}}{\partial \mathbf {B}_{\rm g}} : \left( \mathbf {L}_{\rm g}\mathbf {B}_{\rm g} + \mathbf {B}_{\rm g}\mathbf {L}_{\rm g}^{T}\right) + \mathcal {S}_0 \ge 0. }$

 If we furthermore let $\psi _{\rm e}$ and $\psi _{\rm g}$ only depend on the invariants of $\mathbf {C}_{\rm e}$ and $\mathbf {B}_{\rm g}$, their derivatives $\frac{\partial \psi _{\rm e}}{\partial \mathbf {C}_{\rm e}}$ and $\frac{\partial \psi _{\rm g}}{\partial \mathbf {B}_{\rm g}}$ are symmetric and commute with either $\mathbf {C}_{\rm e}$ or $\mathbf {B}_{\rm g}$. Combining this with the properties of the double contracting product, the reduced Clausius–Duhem inequality can be written only in terms of the symmetric part $\mathbf {D}_{\rm g} = {\text {sym}}\mathbf {L}_{\rm g}$ of the growth velocity gradient, i.e.

11 $$\begin{aligned} \dot{\gamma }_{\rm red} = \left[ \mathbf {M} - \varvec{\chi }\right] : \mathbf {D}_{\rm g} + \mathcal {S}_0 \ge 0, \end{aligned}$$

where the Mandel stress tensor is denoted by ${\mathbf {M} = 2\mathbf {C}_{\rm e}\frac{\partial \psi _{\rm e}}{\partial \mathbf {C}_{\rm e}}}$ and the back-stress tensor is given as ${\varvec{\chi } = 2\mathbf {B}_{\rm g}\frac{\partial \psi _{\rm g}}{\partial \mathbf {B}_{\rm g}}}$. Similar to classical plasticity theory, see e.g. (Vladimirov et al. 2008), one can identify the difference of the Mandel stress tensor $\mathbf {M}$ and the back-stress tensor $\varvec{\chi }$ as the conjugated driving force for the evolution of growth. It is therefore natural to describe the evolution equation for $\mathbf {D}_{\rm g}$ in terms of these quantities. Notice that $\mathbf {M}$ and $\varvec{\chi }$ are located within a grown intermediate configuration, where they can be clearly identified as stress like quantities. This becomes clear by the fact that $\mathbf {M}$ has the same invariants as the Kirchhoff stress tensor $\varvec{\tau }$ and, thus, has a clear physical meaning (see “Appendix 1.2”). Pulling $\mathbf {M}$ and $\varvec{\chi }$ back to the reference configuration will yield a loss of such clear physical interpretation. Nevertheless, from a conceptual and computational point of view, a pullback of these quantities to the reference configuration is desirable (for details see e.g. Dettmer and Reese 2004 and Vladimirov et al. 2008). Taking into account the relation $\mathbf {D}_{\rm g} = \frac{1}{2}\mathbf {F}_{\rm g}^{-T}\dot{\mathbf {C}}_{\rm g}\mathbf {F}_{\rm g}^{-1}$ one can rewrite the Clausius–Duhem inequality purely in terms of quantities located within the reference configuration, i.e.

12 $$\begin{aligned} \begin{aligned} \dot{\gamma }_{\rm red}&= \left( \mathbf {F}_{\rm g}^{-1}\mathbf {M}\mathbf {F}_{\rm g}^{-T} - \mathbf {F}_{\rm g}^{-1}\varvec{\chi }\mathbf {F}_{\rm g}^{-T}\right) : \frac{1}{2}\dot{\mathbf {C}}_{\rm g} + \mathcal {S}_0\\&= \left( \mathbf {\Gamma } - \mathbf {X}\right) : \frac{1}{2}\dot{\mathbf {C}}_{\rm g} + \mathcal {S}_0\\&= \mathbf {\Sigma }: \frac{1}{2}\dot{\mathbf {C}}_{\rm g} + \mathcal {S}_0 \ge 0. \end{aligned} \end{aligned}$$

Similar to the formulation given with respect to the grown intermediate configuration, it is reasonable to define the evolution of the growth-related right Cauchy–Green tensor $\mathbf {C}_{\rm g}$ in terms of the thermodynamically conjugated driving forces $\mathbf {\Gamma } = \mathbf {C}_{\rm g}^{-1}\mathbf {C}\mathbf {S}$ and $\mathbf {X}=2\frac{\partial \psi _{\rm g}}{\partial \mathbf {C}_{\rm g}}$. It is important to mention that using $\mathbf {C}_{\rm g}$ as the internal variable yields the fact that $\mathbf {F}_{\rm g}$ must never be computed in the first place. Such an approach is in clear contrast to the standard formulations in volumetric growth modelling, where the growth tensor itself is usually explicitly prescribed.

### Evolution of growth

Up to this point, the framework presented is very general and could be used to describe a wide variety of inelastic phenomena in finite deformations. It is therefore the choice of evolution equations for $\mathbf {C}_{\rm g}$ that explicitly defines a particular kind of inelastic material model. For the most simple modelling assumption of a purely isotropic growth response, the inelastic part of the deformation gradient is usually defined as $\mathbf {F}_{\rm g} = \vartheta \mathbf {I}$, where $\vartheta$ describes the growth induced stretch (see e.g. Lubarda and Hoger 2002; Himpel et al. 2005; Göktepe et al. 2010). Using the thermodynamic framework described above, this assumption naturally leads to an evolution equation of $\mathbf {C}_{\rm g}$, which can be written as

13 $$\begin{aligned} \dot{\mathbf {C}}_{\rm g} := 2\frac{\dot{\vartheta }}{\vartheta }\mathbf {C}_{\rm g}. \end{aligned}$$

Within this context, a scalar valued evolution equation ${\dot{\vartheta } = f(\vartheta , \mathbf {M}, \varvec{\chi }, ...)}$ is used to determine the overall growth response (see “Appendix 1.3” for a more detailed example). Although the a priori assumption of $\mathbf {F}_{\rm g}$ being a diagonal tensor is tempting due to its computational simplicity, it was already pointed out in various publications that such an assumption is not reasonable for many applications (see e.g. Soleimani et al. 2020; Braeu et al. 2019, 2017). This is especially the case for scenarios in which the body cannot grow freely but is restricted by complex boundary conditions. To overcome this issue, a new volumetric growth model is proposed in the following.

#### Finite growth using a growth potential

**Fig. 2:**
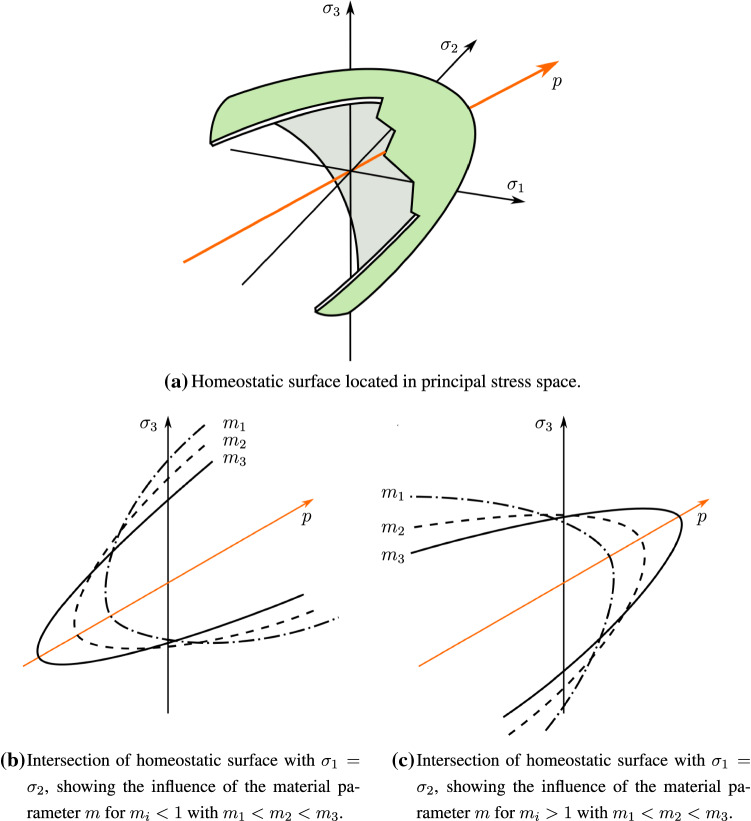
Schematic representation of the homeostatic surface defined by Eq. (18) displayed in principal stress space. The hydrostatic axis $p = {\text {tr}}\left( \mathbf {M}- \varvec{\chi }\right)$ is shown in orange. The eigenvalues of $\mathbf {M} - \varvec{\chi }$ are denoted by $\sigma _i$

As described in the introduction, cell-mediated expansion or compaction of engineered tissues takes place in such a way that a preferred homeostatic stress state is reached within the material. In the present work, it is assumed that this homeostatic state can be described in terms of a scalar equivalent stress. Thus, growth always takes place, if this equivalent stress is not equal to the preferred stress state of the biological material. These considerations lead us to the introduction of a general growth potential

14 $$\begin{aligned} \Phi := \tilde{\Phi }\left( \mathbf {M}, \varvec{\chi }, \alpha _1, ..., \alpha _n\right) , \end{aligned}$$

which is a function of the conjugated driving forces as well as a set of material parameters $\alpha _i$. Similar to the representation used in classical plasticity theory, this potential can be represented as a surface, located within the principal stress space, which contains all eligible homeostatic stress states. It will therefore be named *homeostatic surface* in the following. An example for such a homeostatic surface can be found in Fig. 2. The overall goal of this process is to approach $\Phi = 0$ over time and therefore reach a stress state that lies on the homeostatic surface. Furthermore, it seems natural that such growth processes always try to minimize the amount of energy needed to reach the homeostatic state. Hence, the direction of growth response will be described by the derivative of the growth potential, i.e. $\mathbf {N} = \frac{\partial \Phi }{\partial \mathbf {M}}$. It is furthermore obvious that homeostasis is never reached instantaneously but rather approached over a certain period of time. To account for this temporal effect, we introduce the growth multiplier $\dot{\lambda }_{\rm g} := \dot{\lambda }_{\rm g}\left( \Phi , \eta , \beta _1, ..., \beta _n\right)$ defined as an explicit function of the growth potential, the growth velocity $\eta$ and a set of material parameters $\beta _i$. Subsequently, the considerations above lead us to an associative growth evolution law that is postulated as

15 $$\begin{aligned} \mathbf {D}_{\rm g} := \dot{\lambda }_{\rm g} \frac{\mathbf {N}}{||\mathbf {N}||}. \end{aligned}$$

In general, we do not want to restrict the choice of $\Phi$ to only positive homogeneous potentials of degree one. This has the side effect that $||\mathbf {N}|| = 1$ cannot be guaranteed, which yields the need to normalize the growth direction tensor to assure that only $\dot{\lambda }_{\rm g}$ has an influence on the amount of accumulated growth deformations. As before, we furthermore can define the given evolution equation in terms of quantities located purely within the reference configuration. To achieve this, a pullback operation is performed that yields

16 $$\begin{aligned}\dot{\mathbf {C}}_{\rm g} = \frac{2\dot{\lambda }_{\rm g}}{||\mathbf {N}||} \mathbf {F}_{\rm g}^{T}\mathbf {N}\mathbf {F}_{\rm g} = \dot{\lambda }_{\rm g}\mathbf {f} = \dot{\lambda }_{\rm g}\mathbf {g}\mathbf {C}_{\rm g}, \end{aligned}$$

including the general second order tensors $\mathbf {f}=\frac{2}{||\mathbf {N}||}\mathbf {F}_{\rm g}^{T}\mathbf {N}\mathbf {F}_{\rm g}$ as well as $\mathbf {g} = \mathbf {f}\mathbf {C}_{\rm g}^{-1}$.

##### *Remark*

The same result for Eq. (16) could also be obtained following (Reese et al. 2021) and the procedures proposed therein. Therefore, this evolution law could be interpreted in the broader context of a theory describing the evolution of general structural tensors.

Since this approach is very similar to the classical models of visco-plasticity, the attentive reader may ask how far these approaches differ. In the case of plasticity, the yield criterion is used to clearly distinguish between the purely elastic and elasto-plastic state, i.e. the yield criterion must always be less than or equal to zero. In contrast, the growth potential $\Phi$ does not serve to distinguish between an elastic and inelastic region, since an ‘elasto-growth’ state is present for both $\Phi < 0$ and $\Phi > 0$. Only in case of $\Phi = 0$ no further growth has to take place, since homeostasis has already been reached. This behaviour is also reflected by the growth multiplier, which in contrast to plasticity can also have negative values. In the authors opinion, this modelling approach has several advantages: (1) As stated earlier, the direction of growth does not have to be prescribed a priori, (2) the complexity of the material model is reduced and (3) due to the strong similarities to plasticity, one can rely on a large repertoire of knowledge from this field, both from a modelling and numerical point of view. For instance, one could argue that the preferred stress can not only be described by only one smooth growth potential. Having e.g. the concept of multisurface plasticity in mind, it would be easy to adopt the growth potential by a more sophisticated approach. In addition, it is also possible, for instance, to take into account a changing preferred stress using an approach similar to the concept of isotropic hardening.

##### *Remark*

It is important to point out that while the model developed here is strongly inspired by the methods of classical plasticity theory, the micromechanical interpretations of these purely phenomenological approaches do not correspond to each other in any way. Furthermore, it is important to note that in reality, instead of a sharply defined homeostatic state, a fuzzy state or possibly even a multitude of such states might occur.

Before defining a specific form of the growth potential, we first take a closer look at the structure of such a potential. It has already been pointed out above that it is reasonable to assume that growth in biological tissues tends to be of isotropic nature only in the absence of restricting boundary conditions. This idea leads us to the definition of the growth potential as a function of the volumetric invariant $I_1 := {\text {tr}}\left( \mathbf {M}-\varvec{\chi }\right) = {\text {tr}}\left( \mathbf {\Sigma }\mathbf {C}_{\rm g}\right)$. To allow also for an anisotropic growth response, we furthermore include the deviatoric invariant $J_2 := \frac{1}{2}{\text {tr}}\left( {\text {dev}}\left( \mathbf {M}-\varvec{\chi }\right) ^2\right) = \frac{1}{2}{\text {tr}}\left( {\text {dev}}\left( \mathbf {\Sigma }\mathbf {C}_{\rm g}\right) ^2\right)$ (see “Appendix 1.4”), where we use the deviatoric projection given as ${{\text {dev}}\left( \bullet \right) =\left( \bullet \right) -\frac{1}{3}{\text {tr}}\left( \bullet \right) \mathbf {I}}$. With these considerations at hand, we propose a general form for the growth potential as

17 $$\begin{aligned}&\Phi := \Phi \left( I_1, J_2, \omega _{\rm hom}\right) \nonumber \\&\quad = \phi _1\left( I_1\right) + \phi _2\left( J_2\right) - \omega _{\rm hom}. \end{aligned}$$

Here, the material parameter $\omega _{\rm hom}$ describes a stress like quantity defining the state of homeostasis. It is important to emphasize that the combination of $I_1$ and $J_2$ is crucial for the proposed material model. If the potential was merely defined in terms of the volumetric invariant $I_1$, the growth direction tensor would become proportional to the identity tensor which consequently yields an evolution equation that is similar to the isotropic evolution law given in Eq. (13). It is the dependency on $J_2$ that introduces an anisotropic growth behaviour, since the growth direction tensor no longer necessarily has to correspond to the identity. Nevertheless, in case of purely volumetric stress states, the dependency on $I_1$ ensures the desired isotropic growth response. This consideration yields an exclusion of any purely deviatoric potential, e.g. of von Mises-type potentials. Furthermore, any suitable potential must fulfil $\frac{\partial \Phi }{\partial \mathbf {M}} \ne \mathbf {0}$ for all $\left( \mathbf {M}-\varvec{\chi }\right) \in \left( \mathbb {R}^3\times \mathbb {R}^3\right)$ in order to guarantee a well-defined growth direction for any arbitrary loading condition.

#### Choice of the growth potential and growth multiplier evolution

The form of a specific potential depends strongly upon the needs of the given application. Unfortunately, there is currently a lack of meaningful experimental data regarding the mechanics of volumetric growth. We therefore choose a potential that proofed to be able to predict our macroscopical observations and further satisfies the general requirements stated above. For this purpose, the quadratic potential as described e.g. by Stassi-D’Alia (1967) and Tschoegl (1971) is used in the following. This potential can be expressed in terms of $\omega _{\rm hom} = m\sigma _{\rm g}^2$ including the material parameters $m$ and $\sigma _{\rm g}$, i.e.

18 $$\begin{aligned} \Phi = 3J_2 - (1-m)\sigma _{\rm g} I_1 - m\sigma _{\rm g}^2. \end{aligned}$$

As shown in Fig. 2a, the homeostatic state defined by this particular growth potential forms a hyperbolic surface within the principal stress space. The tipping point of this parabola is located on the hydrostatic axis, where its precise location is determined by the parameter $m$ (see Fig. 2b, c). From Eq. (18) it is obvious that both parameters must always be greater than zero. It is furthermore important to notice that the opening side of the parabolic potential lies within the compressive regime for $m< 1$ and in the tensional regime for $m> 1$, respectively. Since a choice of $m=1$ describes a von-Mises-type model such a choice of this parameters must be avoided. It is worth noticing from Fig. 2 that this particular form of the growth potential leads to a different material response in the compressive and tensional regime, respectively. Using this form of the growth potential yields the growth direction tensor as

19 $$\begin{aligned} { \mathbf {N} = 3{\text {dev}}\left( \mathbf {M}-\varvec{\chi }\right) - \left( 1-m\right) \sigma _{\rm g}\mathbf {I}. } \end{aligned}$$

It is important to notice that this quantity can be reformulated with respect to quantities located within the reference configuration by using the identities from “Appendix 1.4”. In this case, Eq. (16) can be written solely in terms of $\mathbf {C}_{\rm e}$ since ${\mathbf {F}_{\rm e}^{T}\mathbf {N}\mathbf {F}_{\rm e}} = \mathbf {C}_{\rm e}\left( 3{\text {dev}}\left( \mathbf {\Sigma }\mathbf {C}_{\rm e}\right) - \left( 1-m\right) \sigma _{\rm e}\mathbf {I}\right)$. To complete the set of equations needed to describe the evolution of the growth-related right Cauchy–Green tensor, we furthermore define a particular form for the evolution of the growth multiplier $\dot{\lambda }_{\rm e}$. From a physically motived point of view, it seems natural that the growth response increases with the deviation of the current stress state from homeostasis. We therefore assume the change in accumulated growth stretch is proportional to the current value of the growth potential. This furthermore ensures that the growth process stops as soon as homeostasis is reached. With these assumptions in mind, we choose the well-established approach proposed in Perzyna (1966) and Perzyna (1971), i.e.

20 $$\begin{aligned} \dot{\lambda }_{\rm e} := \frac{1}{\eta }\left( \frac{\Phi }{m\sigma _{\rm e}^2}\right) ^{\frac{1}{\nu }}. \end{aligned}$$

Herein the growth multiplier is defined in terms of the growth relaxation time $\eta$ as well as a nonlinearity parameter $\nu$.

#### Choice of Helmholtz free energy

Until this point, the constitutive framework presented herein has been described without defining a particular form of the Helmholtz free energy. In general, the choice of the energy potential depends upon the specific type of material one would want to model. For the time being, we choose a compressible Neo-Hookean-type model to describe the elastic response of the material. Therefore, the elastic energy $\psi _{\rm e}$ is written in terms of the Lamé constants $\mu$ and $\Lambda$ as

21 $$\begin{aligned}\psi _{\rm e} &= \frac{\mu }{2}\left( {\text {tr}}\mathbf {C}_{\rm e} - 3\right) - \mu {\text {ln}}J_{\rm e} + \frac{\Lambda }{4}\left( J_{\rm e}^2 - 1 - 2{\text {ln}}J_{\rm e}\right) . \end{aligned}$$

Following the argumentation in Sect. 2.2, we furthermore define the growth-related Helmholtz free energy $\psi _{\rm e}$ in terms of a stiffness like material parameter $\kappa _{\rm e}$ such that

22 $$\begin{aligned} \psi _{\rm e} = \frac{\kappa _{\rm e}}{2}\left( J_{\rm e}^2 - 1 - 2{\text {ln}}J_{\rm e}\right) . \end{aligned}$$

This particular choice of the growth-related energy obviously fulfils the general requirements for the definition of a strain energy density, i.e. $\psi _{\rm e}(J_{\rm e} \rightarrow 0) \rightarrow \infty$ as well as $\psi _{\rm e}(J_{\rm e} = 1) = 0$ and $\psi _{\rm e}(J_{\rm e} \rightarrow \infty ) \rightarrow \infty$. With these definitions at hand, the second Piola–Kirchhoff stress tensor and the back-stress tensor can be derived as

23 $$\begin{aligned} \begin{aligned} \mathbf {S}&= \mu \left( \mathbf {C}_{\rm e}^{-1} - \mathbf {C}^{-1}\right) + \frac{\Lambda }{2}\left( \left( \frac{J}{J_{\rm e}}\right) ^2 - 1\right) \mathbf {C}^{-1}\\ \mathbf {X}&= \kappa _{\rm e}\left( J_{\rm e}^2-1\right) \mathbf {C}_{\rm e}^{-1} \end{aligned} \end{aligned}$$

Notice that the conjugated driving force $\mathbf {\Gamma }$ can be easily computed, if $\mathbf {C}_{\rm e}$ and $\mathbf {S}$ are known (see Sect. 2.3).

## Numerical implementation

To incorporate the volumetric growth model at hand into a finite element simulation framework, a suitable time integration technique has to be used for evolution Eq. (16). As shown for example by Weber and Anand (1990), Simo (1992), Reese and Govindjee (1998), Vladimirov et al. (2008) and discussed in further detail by Korelc and Stupkiewicz (2014), the exponential mapping algorithm is a very suitable choice for the treatment of the given evolution equation. We will therefore briefly describe this approach in the following.

Starting with the discrete time increments $\Delta t = t_{n+1} - t_n$, we introduce the growth increment $\Delta \lambda _{{\rm g}_{n+1}} = \Delta t \dot{\lambda }_{\rm g}$ as the discretized version of the growth multiplier. With this at hand, the exponential integration scheme for the evolution Eq. (16) can be written in terms of the general second-order tensors ${\mathbf {g}}$ and ${\mathbf {f}}$ as introduced in context of Eq. (16), e.g.

24 $$\begin{aligned} \mathbf {C}_{{\rm g}_{n+1}} = {\text {exp}}\left( \Delta \lambda _{\rm e}\mathbf {g}\right) \mathbf {C}_{{\rm g}_n}. \end{aligned}$$

Notice that subscript $n+1$ will be dropped in the following for notational simplicity, which means that any discrete quantity without subscript will be associated with the current time step. Following the argumentation within (Vladimirov et al. 2008) and (Dettmer and Reese 2004) Eq. (24) can be reformulated to ensure the symmetry of $\mathbf {C}_{\rm e}$. Furthermore, the authors mentioned above show that the exponential function within this equation can be expressed in terms of the growth-related right stretch tensor $\mathbf {U}_{\rm e} = \sqrt{\mathbf {C}_{\rm e}}$. Consequently, this leads to the discretized evolution equation given as

25 $$\begin{aligned}\mathbf {C}_{{\rm g}_n}^{-1} = \mathbf {U}_{\rm e}^{-1}{\text {exp}}\left( \Delta \lambda _{\rm e}\mathbf {U}_{\rm e}^{-1}\mathbf {f}\mathbf {U}_{\rm e}^{-1}\right) \mathbf {U}_{\rm e}^{-1}. \end{aligned}$$

In order to complete the set of discrete constitutive equations, the discrete growth multiplier $\Delta \lambda _{\rm g}$ must be determined. This can be achieved by reformulating Eq. (20) (see e.g. Simo and Hughes 1998 and de Souza Neto et al. 2008), i.e.

26 $$\begin{aligned} \Phi = m\sigma _{\rm g}^2\left( \Delta \lambda _{\rm g}\eta \right) ^\nu . \end{aligned}$$

Since both of the discrete constitutive equations are nonlinear in their arguments, a local iterative solution algorithm must be applied at integration point level to solve for both, the internal variable $\mathbf {U}_{\rm g}^{-1}$ as well as the growth increment $\Delta \lambda _{\rm g}$. It is convenient for such algorithms to write the evolution equations in terms of a set of coupled residual functions, which read in the case of this material model

27 $$\begin{aligned}\mathbf {r}_{\rm g}&= -\mathbf {C}_{{\rm g}_n}^{-1} + \mathbf {U}_{\rm g}^{-1}{\text {exp}}\left( \Delta \lambda _{\rm g}\mathbf {U}_{\rm g}^{-1}\mathbf {f}\mathbf {U}_{\rm g}^{-1}\right) \mathbf {U}_{\rm g}^{-1} = \mathbf {0}\\ r_\Phi&= \Phi - m\sigma _{\rm g}^2\left( \frac{\Delta \lambda _{\rm g}}{\Delta t}\eta \right) ^\nu = 0. \end{aligned}$$

Due to the symmetry of $\mathbf {U}_{\rm g}$, the tensor valued residual function $\mathbf {r}_{\rm g}$ can be transformed into Voigt notation, which is computationally more efficient than solving the full tensorial equation. When applying a Newton–Raphson procedure to solve Eq. (27), the increments of the equations’ arguments can be found by solving a linearized system of equations, i.e.

28 $$\begin{aligned} \displaystyle \begin{pmatrix} \frac{\partial \hat{\mathbf {r}}_{\rm g}}{\partial \hat{\mathbf {U}}_{\rm g}} &{} \frac{\partial \hat{\mathbf {r}}_{\rm g}}{\partial \Delta \lambda _{\rm g}} \\ \frac{\partial r_\Phi }{\partial \hat{\mathbf {U}}_{\rm g}} &{} \frac{\partial r_\Phi }{\partial \Delta \lambda _{\rm g}} \\ \end{pmatrix} \Delta \begin{pmatrix} \hat{\mathbf {U}}_{\rm g} \\ \Delta \lambda _{\rm g} \end{pmatrix} = -\begin{pmatrix} \hat{\mathbf {r}}_{{\rm g}_n} \\ r_ {\Phi _n} \end{pmatrix}. \end{aligned}$$

During the solution process, these increments are recomputed for every iteration step in which they are used to update the local iteration procedure. The partial derivatives used herein are not computed analytically but rather calculated by means of an algorithmic differentiation approach. For this, the software package *AceGen*, as described e.g. in Korelc (2002) and Korelc (2009), is being used to automatically generate source code for the computation of the tangent operators.

Since the local material response is implicitly included within the global material tangent operator of a finite element simulation, we furthermore need to derive this tangent in a consistent manner. Otherwise, quadratic convergence of the global iteration scheme would not be reached. For this, one should bear in mind that the second Piola–Kirchhoff stress is a function of the right Cauchy–Green tensor as well as the internal variables. Within the given framework, the material tangent operator can be expressed as

29 $$\mathbb{C} = 2\left( {\left. {\frac{{\partial {\mathbf{S}}}}{{\partial {\mathbf{C}}}}} \right|_{{{\mathbf{U}}_{g} }} } \right.{\text{ }} + \left. {\frac{{\partial {\mathbf{S}}}}{{\partial {\mathbf{U}}_{g} }}} \right|_{{\mathbf{C}}} {\text{ }} \left. {:\frac{{\partial {\mathbf{U}}_{g} }}{{\partial {\mathbf{C}}}}} \right).{\text{ }}$$

Similar to the local tangent operator, these partial derivatives are computed using the software package *AceGen*. For this, the partial derivative of the growth-related stretch tensor $\mathbf {U}_{\rm g}$ with respect to the right Cauchy–Green tensor can be determined from the following relation

30 $$\begin{aligned} \Delta \begin{pmatrix} \hat{\mathbf {U}}_{\rm g} \\ \Delta \lambda _{\rm g} \end{pmatrix} = - \begin{pmatrix} \frac{\partial \hat{\mathbf {r}}_{\rm g}}{\partial \hat{\mathbf {U}}_{\rm g}} &{} \quad \frac{\partial \hat{\mathbf {r}}_{\rm g}}{\partial \Delta \lambda _{\rm g}} \\ \frac{\partial r_\Phi }{\partial \hat{\mathbf {U}}_{\rm g}} &{} \quad \frac{\partial r_\Phi }{\partial \Delta \lambda _{\rm g}} \\ \end{pmatrix}^{-1} \begin{pmatrix} \frac{\partial \hat{\mathbf {r}}_{\rm g}}{\partial \hat{\mathbf {C}}} \\ \frac{\partial r_\Phi }{\partial \hat{\mathbf {C}}} \end{pmatrix} \Delta \hat{\mathbf {C}}. \end{aligned}$$

Here, we reuse the fully converged residual and Jacobian from the local solution process. Then, the desired partial derivative is given as the corresponding $6\times 6$ submatrix located in the upper left corner of the right-hand side matrix product.

## Numerical examples

In the following section, numerical examples are presented to examine and discuss various aspects of the material model introduced above. First, we show the influence of boundary conditions on the development of the volumetric growth process using a simple block model. For this purpose, volumetric growth in the absence of geometrically constraining boundary conditions is evaluated as well as the impact of both temporal constant and time-dependent constraining boundary conditions. Next, we investigate the influence of the introduced set of material parameters, before showing structural examples of a shrinking tissue stripe and comparing its growth-related response to an isotropic growth formulation. Finally, we show a qualitative comparison of our model with experimental data from the literature. For the finite element simulations, we implemented the presented material model as well as the element formulation itself into the *FEAP* software package (Taylor and Govindjee 2020) in terms of a *user-element* routine. For meshing and visualization of the structural examples we have used the open source software tools *GMSH* (Geuzaine and Remacle 2009) and *Paraview* (Ahrens et al. 2005). Furthermore, the open source parallelization tool *GNU Parallel* (Tange 2011) was used during evaluations of the examples shown below.

**Table 1:** Material parameters for numerical examples

	$\mu$	$\Lambda$	$\kappa _{\rm g}$	$m$	$\sigma _{\rm g}$	$\eta$	$\nu$
	$\left[ \frac{\text {N}}{\text {mm}^2}\right]$	$\left[ \frac{\text {N}}{\text {mm}^2}\right]$	$\left[ \frac{\text {N}}{\text {mm}^2}\right]$	$\left[ -\right]$	$\left[ \frac{\text {N}}{\text {mm}^2}\right]$	$\left[ \text {s}\right]$	$\left[ -\right]$
Geom. unconstrained growth	40	400	150	1.2	70	20	1.0
Geom. constrained growth	40	400	250	1.2	200	100	1.0
Clamped tissue stripe	100	800	150	2.0	250	100	1.0

### Geometrically unconstrained growth

**Fig. 3:**
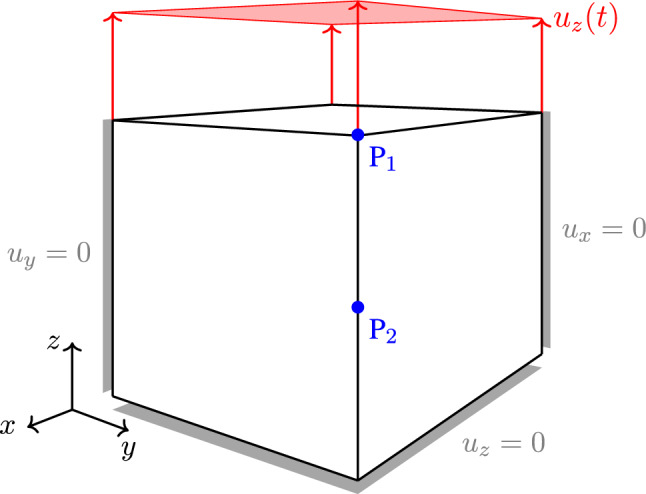
Geometrical block model with uniform side length of $1~\text {mm}$. Uniaxial boundary conditions are given in grey, and time-dependent displacement $u_z(t)$ is denoted in red. Evaluation points $P_1 = (1,1,1)$ and $P_2=(1,1,0.5)$ are given in blue

**Fig. 4:**
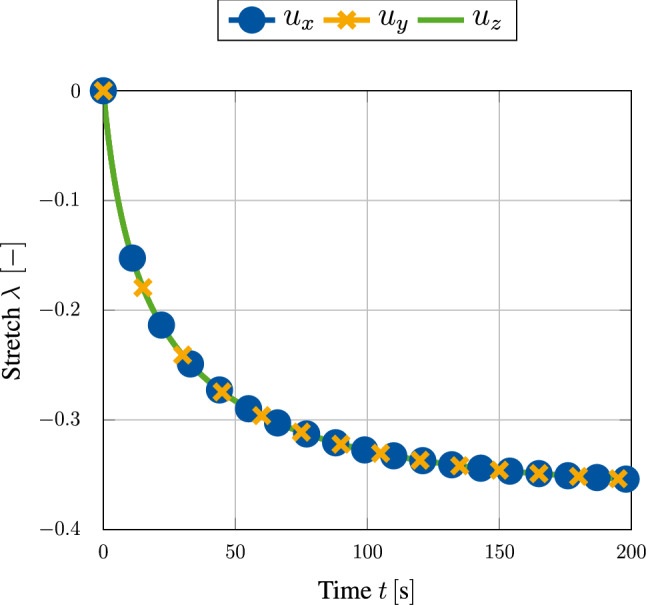
Isotropic growth behaviour resulting in a uniform contraction in all three spatial dimensions. No constraining boundary conditions are applied (i.e. no $u_z(t)$). Stretches are evaluated at point $\text {P}_1$ (see Fig. 3)

**Fig. 5:**
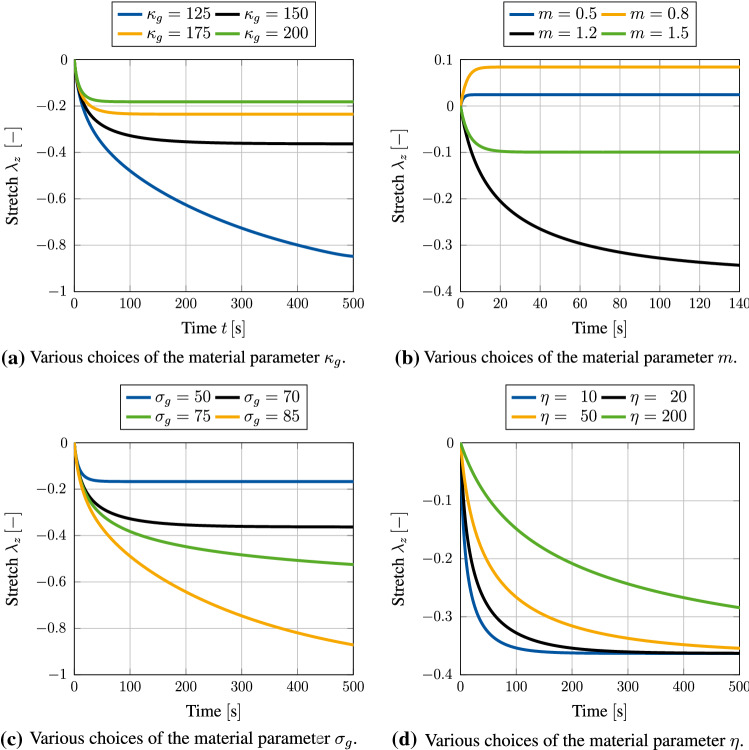
Growth-induced stretch due to contraction of a block specimen for various sets of material parameters. No constraining boundary conditions are applied (i.e. no $u_z(t)$). Stretches are evaluated at point $\text {P}_1$ (see Fig. 3)

As a first example, we use the geometrical model shown in Fig. 3 without applying any time-dependent displacement boundary condition $u_z(t)$. Therefore, the specimen is able to expand or contract freely throughout the whole simulation, which should result in an isotropic growth response. We furthermore use the set of material parameters given in Table 1. The growth response for a choice of $m=1.2$ is visualized in Fig. 4. Shown by the stretches of point $\text {P}_1$ located in the upper corner of the given block geometry, it is obvious that the specimen contracts as expected. Since no constraining boundary conditions are applied, the overall stress within this system should always be equal to zero and therefore could never reach a state of tensional homeostasis. It is the additional growth-related free energy, which leads to the limitation of the otherwise infinite shrinking process. One can observe this influence really well in Fig. 5a, where lower values of $\kappa _{\rm g}$ lead to a more pronounced shrinking of the specimen. It is worth noticing that $\kappa _{\rm g} > 0$ must hold for any simulation, since neglecting the contribution of internal pressures would lead to non-physical behaviour and consequently to an unstable simulation.

It is furthermore shown in Fig. 5d that the growth rate parameter $\eta$ has only an impact on the speed at which the volumetric growth process approaches the desired homeostatic state but not on its magnitude. However, as shown in Fig. 5b, c a change in magnitude of the homeostatic state can be achieved by variation of $m$ and $\sigma _{\rm g}$. As already described in Sect. 2.4.2, the material parameter $m$ defines the location of the growth potential’s tipping point on the hydrostatic axis. For values of $m<1$ this point lies in the compressive regime, while a choice of $m>1$ pushes this point into the tension regime. As a result, the specimen approaches homeostasis either by expansion or by shrinkage. This behaviour is really well reflected within Fig. 5b. It is furthermore important to point out that for a choice of $m=1$ the homeostatic potential introduced in Eq. (18) becomes a *von-Mises*-type criterion, which must not be applied due to its purely deviatoric nature. Therefore, this particular choice of $m$ should be avoided when using the potential introduced above.

### Geometrically constrained growth

For the next example, we choose a stepwise time-dependent displacement $u_z(t)$ to which the block given in Fig. 3 is subjected. For the first $250$ time steps, the displacement is held constant at $u_z(t) = 0~\text {mm}$ before being raised to $u_z(t)=0.3~\text {mm}$ and held constant for another $200$ time steps. Next, we apply compression by setting $u_z(t)=-0.1~\text {mm}$ and holding it constant for another $250$ time steps. At last, $u_z(t)$ is reset to zero again. The material parameters for this example are given in Table 1.

**Fig. 6:**
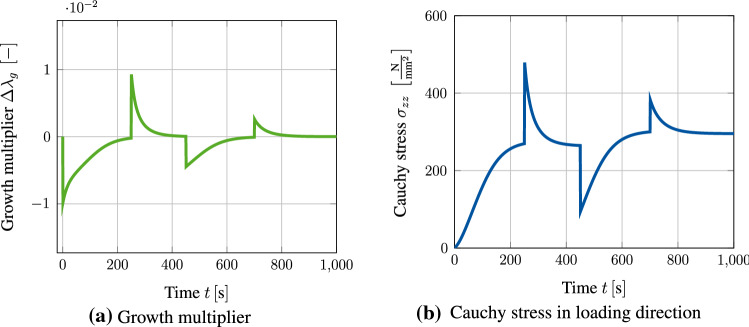
Evolution of Cauchy stress $\sigma _{zz}$ and growth multiplier $\Delta \lambda _{\rm g}$ during stepwise loading of block specimen with $u_z(t)$. Both quantities are evaluated at point $\text {P}_1$ (Fig. 3). Right: The stress response is always converging towards a homeostatic state. This state is slightly different, after coming out of the compressive regime. This can be explained by the accumulated internal pressures described by the energy $\psi _{\rm g}$. Left: Growth multiplier indicating that the specimen is either expanding or shrinking to reach homeostasis

When applying this stepwise alternating stretch to the given block specimen, it can be seen in Fig. 6 that the material shrinks and expands depending on the current loading state, respectively. During the first loading period, the accumulated Cauchy stress $\sigma _{zz}$ rises to a value of approximately $300~\text {MPa}$, which is due to a contraction induced by the volumetric growth process. This effect is represented by the evolution of the growth multiplier as shown in Fig. 6a. Since the multiplier is negative, the specimen approaches homeostasis by shrinking. Once the displacement is raised to $u_z(t)=0.3~\text {mm}$, the Cauchy stress $\sigma _{zz}$ also rises abruptly before decaying and approaching the same homeostatic stress state as before. This kind of stress reduction is achieved by an expansion of the specimen, which is represented by a positive value of the growth multiplier. The following compression of the specimen causes a negative jump in the overall stress response. This again induces shrinkage of the specimen in order to regain the homeostatic state of approximately $300~\text {MPa}$. It is important to notice that this homeostatic state is slightly higher than the state reached in the loading cycles before. This change is due to the accumulated internal pressures described by the growth-related energy $\psi _{\rm g}$. Consequently, this results in a shift of the homeostatic surface similar to kinematic hardening in plasticity. To what extent this effect corresponds to experimental studies is still unclear due to the lack of available data. However, there is no question that this effect can be adapted to any experimental data without further problems by extending the model, e.g. by a nonlinear formulation. When setting $u_z(t) = 0~\text {mm}$ in the last loading cycle, the Cauchy stresses overshoot this new homeostatic state slightly. This again results in an expansion of the specimen in order to release the excessive stresses.

### Growth of a clamped tissue stripe

**Fig. 7:**
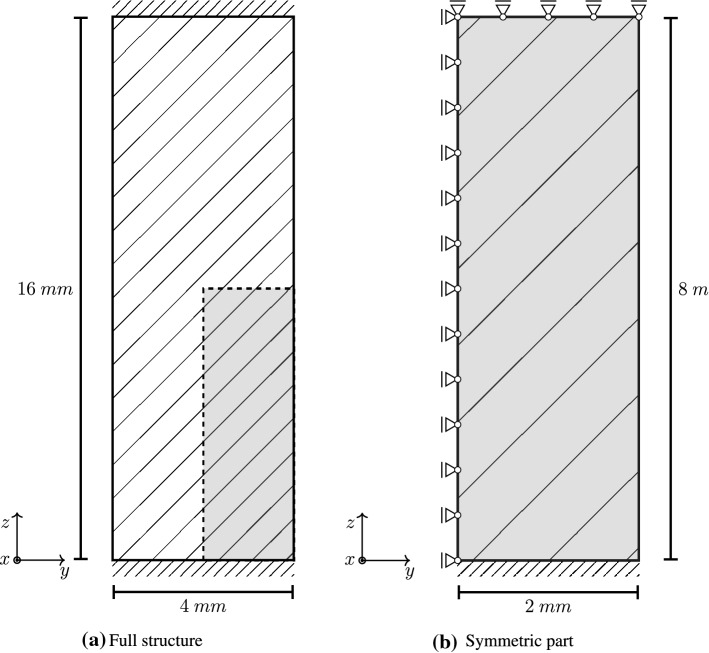
Geometric model of clamped tissue stripe with thickness of $t=2~\text {mm}$. The overall structure is also supported in the $x$ direction

**Fig. 8:**
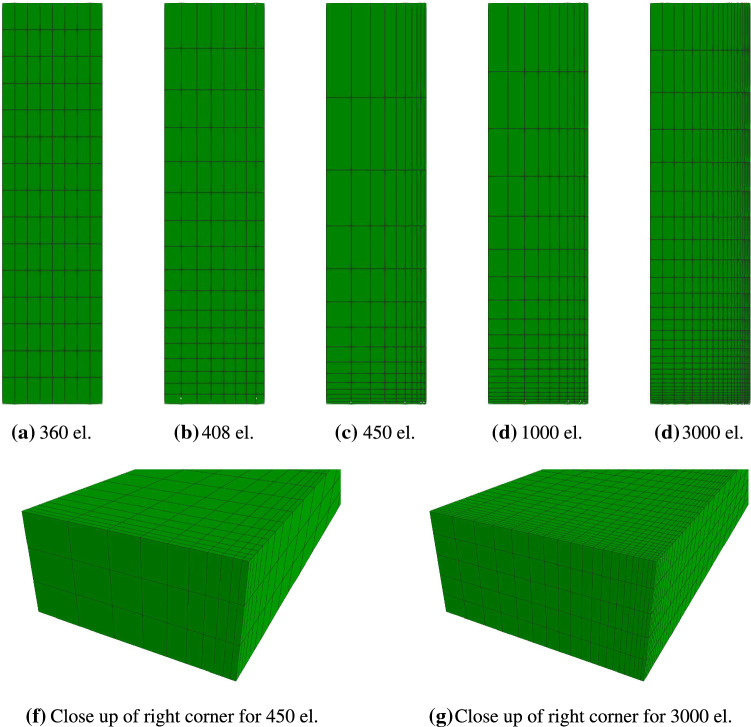
Mesh refinements for symmetric part of clamped tissue stripe

In the next example, we consider the volumetric growth process within a tissue stripe that is clamped at both ends such that no stresses are induced at time $t=0$. Under these conditions, the tissue stripe is expected to shrink, which induces a homeostatic stress state that is dominated by tension. Such effects have been shown experimentally e.g. by Ghazanfari et al. (2015) among others. As illustrated in Fig. 7, symmetric properties are exploited such that only a quarter of the full specimen is used for the following simulations. The elastic and growth-related material parameters applied in this example are chosen in such a way that the desired shrinkage of the specimen is achieved. These parameters are given in Table 1. For the spatial discretization, a standard linear (*Q*1) finite element formulation is adopted with various meshes containing $360$, $408$, $450$, $1000$ and $3000$ elements (see Fig. 8). Since the most pronounced stresses are expected to occur in the lower right corner of the symmetric specimen, the mesh is refined with a focus on this particular region.

**Fig. 9:**
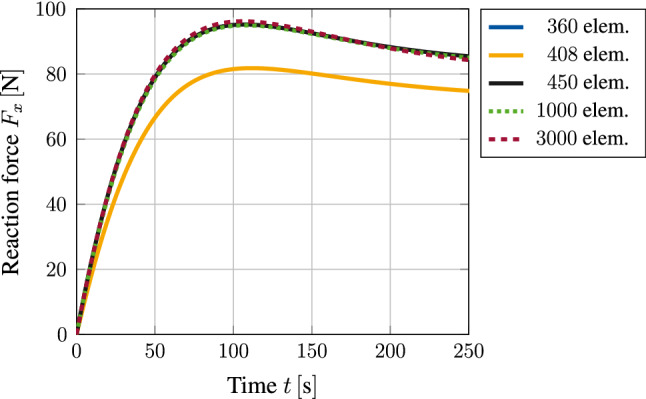
Reaction force of a clamped tissue stripe evaluated at $z=0$ for various mesh sizes. Mesh convergence can be observed nicely

When considering the reaction force $F_x$ evaluated over time at $z=0$, Fig. 9 shows good convergence behaviour for increasing number of elements within the mesh. Similar results can be obtained when evaluating the reaction forces in $y$ and $z$ direction, respectively. Although the solution of a mesh containing $450$ elements has already reached convergence, for visualization purposes, the finest discretization containing $3000$ elements is used in the following.

To show the capabilities of the newly introduced material model, we next compare its response to the growth behaviour of a well-established model for isotropic volumetric growth. For this, we adapted the model of Lubarda and Hoger (2002) such that it is capable of reaching a prescribed homeostatic state. Details about the evolution equations for this particular model are given in “Appendix 1.3”. Within this formulation, we use the material parameter $M_{\rm crit}=80~\text {MPa}$ to describe the homeostatic stress state that shall ultimately be reached. For the positive and negative growth velocities $k^+=0.1$ and $k^-=0.1$ are chosen, respectively. The upper and lower growth boundaries are set to $\vartheta ^+ = 2.0$ and $\vartheta ^-=0.25$, while the shape factors are given as $\gamma ^+=2$ and $\gamma ^-=3$.

**Fig. 10:**
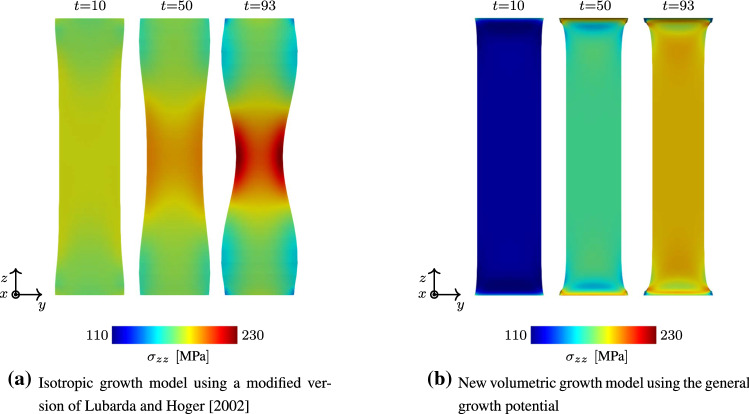
Comparison of an isotropic growth model with the newly introduced formulation. The response of a clamped tissue stripe differs significantly in both, shape as well as the displayed stress response (Cauchy stresses $\sigma _{zz}$)

**Fig. 11:**
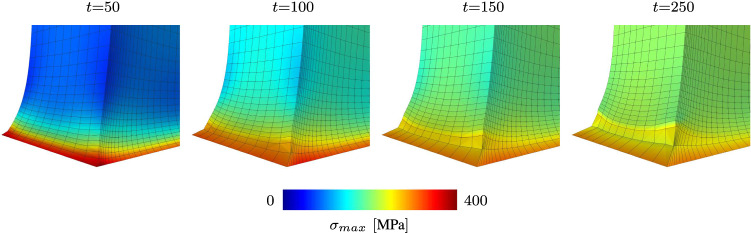
Pronounced distortion of elements at the clamped corner due to growth-related reduction of exceeding stresses. Maximum principal Cauchy stresses are plotted for four different snapshots in time

First of all it is important to notice that the given isotropic formulation shows severe stability problems for the example at hand. More precisely, as soon as material parameters are chosen such that a similar homeostatic stress state shall be reached within the specimen, the simulation becomes unstable after a finite number of time steps and eventually breaks. When taking a closer look at the evolution of the growth process as it is shown for three distinct time steps in Fig. 10, it is obvious that the starting point of the instability can be located at the clamping of the tissue stripe. Due to the initial contraction of the overall tissue stripe, a multi-axial stress state is induced at the clamping. In this region, the stress state soon exceeds the desired homeostatic state which yields an expansion of the material in order to release excessive stresses. While the newly derived growth model reduces this stress state by expanding anisotropically, the isotropic formulation seems not to be able to deal with this effect. This is due to the fact that an isotropic growth formulation can only predict expansion or shrinkage uniformly in all three spatial dimensions. Such a uniform expansion at the foot of the specimen results in a passive compression of the specimen’s middle part, reducing the overall stress within this region and therefore inducing further contraction. This again triggers an increasing expansion in the foot of the specimen. A vicious cycle is born, which eventually leads to the hourglass like shape of the specimen as it is shown in Fig. 10a. Ultimately, this leads to instabilities and a failing simulation at $t = 93$. For sure, it is possible to reduce such unwanted behaviour by variation of the material parameters. Nevertheless, the general problem of a non-physical expansion in the foot area could not be cured with such an approach. This example shows clearly how restrictive and, therefore, unsuitable the assumption of isotropic growth is, even for a relatively simple structure as the one shown in this example. Taking a closer look at the stress response of the newly derived model, one can observe the exceeding maximum principal Cauchy stresses $\sigma _{\max}$ located at the clamped foot of the specimen (see Fig. 11) being released due to the anisotropic expansion process. This effect can also be observed in Fig. 9, where the reaction forces reach a maximum at time $t=100$ and decrease afterwards to approach a converged state. Unfortunately, this effect also leads to a pronounced distortion of the associated elements within the corners of the clamped stripe. Figure 11 shows that this artefact is even noticeable for the finest mesh evaluated. Nevertheless, it is important to emphasize that this effect so far does not have an influence on the stability of the given simulation. Due to the incompressible nature of the material, it is possible that such behaviour is also amplified by shear or volumetric locking effects and would not occur in such a pronounced manner if locking would not play a role. However, the influence of possible locking effects is out of scope for this work.

### Comparison with experimental data

Next, we are comparing our newly derived model to experimental data for the growth response of a clamped engineered tissue stripe. As an experimental reference, we are using the data published just recently in Eichinger et al. (2020), which was kindly provided to us by the authors. In this study, the authors used a cell seeded collagen gel to create the test specimens. For the experiment, those specimens are clamped tension-free at the ends and are cultivated for 27 h within a nutrient solution. After 17 h, a positive or negative perturbation of the measured reaction force in longitudinal direction is applied. The displacement achieved by this perturbation is kept constant in the following course of the experiment. For our simulations, we used a geometric representation of the clamped tissue stripe that is similar to the example shown in Fig. 8 but has a width, height and thickness of $10~\text {mm}$, $60~\text {mm}$ and $2~\text {mm}$. We again used only the symmetric part of the specimen in order to reduce computational effort. Since the microstructure of the collagen gel is somehow similar to polymeric materials, we decided to exchange the elastic strain energy density $\psi _{\rm e}$ in order to better capture the stress stiffening behaviour that can be observed within the experimental data. Here we used the well-known formulation of Arruda and Boyce (1993) (see “Appendix 1.5” for details on the form of the energy).

**Fig. 12:**
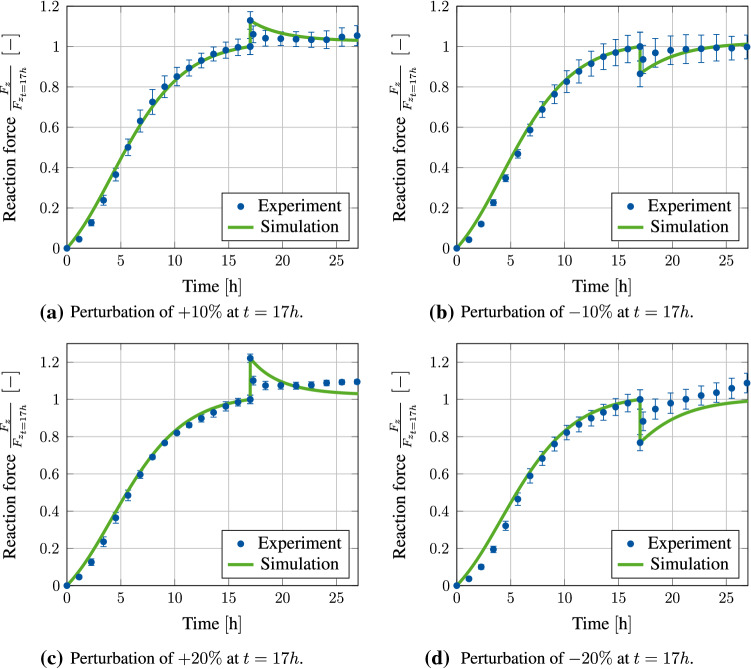
Comparison of simulation results with experimental data of an initially tension-free clamped tissue stripe that is perturbed at time $t=17\text {h}$ with $\pm 10\%$ and $\pm 20\%$ of the homeostatic reaction force measured at this point. Experimental data are plotted as the mean value of all experiments with error bars denoting the SEM [taken with permission from Eichinger et al. (2020)]

Figure 12 shows a comparison of the measured normalized reaction force in longitudinal direction versus the results gained from our simulation. A perturbation of $\pm 10\%$ of the homeostatic reaction force at time $t=17\text {h}$ is shown in Fig. 12a, b, respectively. Here, it is clearly visible that the simulation is close to the experimental results both before and after perturbation and is mostly within the error tolerance. In particular, it can be observed in 12a that both the experiment and the simulation strive towards a new, somewhat higher homeostatic state after the perturbation. In contrast, the homeostatic state in Fig. 12b settles back in approximately the same range as before, which also fits the behaviour observed in the experiment. Figure 12c, d shows the results for a perturbation of $\pm 20\%$. Here, with the material data we use, the results of the experiment are also very well matched up to the point of perturbation. Only after that the simulation results do deviate quantitatively from the measured data. Here, it is particularly noticeable that a higher gradient is achieved in the result curve of the experiment directly after the perturbation. This leads to a faster convergence towards the new homeostatic state. This deviation could possibly be related to the fact that we assume a constant growth rate parameter $\eta$ in our model. However, it cannot be ruled out that this parameter itself should be dependent on other constitutive variables such as the driving force $\left( \mathbf {M}-\varvec{\chi }\right)$. Overall, however, it can be stated that the simulation results represent the experimental data very well in a qualitative sense.

## Conclusion and outlook

In this paper, we developed a novel model for the description of stress-driven volumetric growth. This approach is based on the well-established multiplicative split of the deformation gradient into an elastic and a growth related part. Furthermore, we made the assumption that the given material adapts to its surroundings such that a certain homeostatic stress state is induced within the material. For this homeostatic state, we assume that it can be described in terms of a scalar valued stress like quantity, which led us to the definition of a growth potential. With this idea in hand, we defined an evolution law for the growth related right Cauchy–Green tensor by means of a time-dependent associative rule. This approach is similar but not identical to those often used in the field of finite visco-plasticity. To overcome the issue of infinite growth response, we made use of a similar idea as Braeu et al. (2019) and introduced an additional energy contribution that subsequently counteracts the growth process. In contrast to the latter approach, we use the inelastic part of the volume change, which leads us to a formulation similar to that of kinematic hardening. With these basic modelling assumptions, we were able to show that this approach is capable of simulating both, isotropic and anisotropic growth behaviour within one singular formulation. The distinction between isotropic and anisotropic response is merely a question of the applied boundary conditions and not a-priori prescribed by the structure of the growth tensor. The advantages of this approach have been shown by comparing it to a standard formulation of isotropic growth. In the authors’ opinion, the results of the evaluations shown within this publication are very promising. We furthermore were able to show that our simulations are able to reproduce experimental results published in Eichinger et al. (2020) to a reasonable extend. Since the overall framework of the model is quiet general, it seems possible to easily adapt the growth behaviour to fit various experiments. For this, the choice of alternative descriptions for the growth potential as well as the evolution equation for the growth multiplier could be investigated. To this point, our formulation makes use of a purely isotropic elastic ground model, i.e. Neo-Hooke. Since biological tissue by its very own nature is composed of various components, such as collagen and elastin, the assumption of material isotropy is not ideal. Therefore, we suggest that the given elastic ground model could be extended to also capture the anisotropic nature of the underlying material response properly. This could be achieved by introducing an additional dependency within the Helmholtz free energy that is defined by means of structural tensors describing e.g. the direction of collagen fibres. Furthermore, the investigation of locking effects triggered by the nearly incompressible material behaviour of biological tissues might also be of interest. Since standard low-order finite element formulations are particularly vulnerable in this area, the finite element implementation should therefore be considered more closely. Investigating the influence of reduced integration finite elements seems to be of high benefit. Especially the element formulations Q1SP (see Reese 2005) or Q1STx (see Schwarze and Reese 2011; Barfusz et al. 2021) could improve the computation in terms of computational accuracy as well as computational speed.

## Data Availability

The generated data are stored redundantly and will be made available on demand.

## Appendix 1

### Appendix 1.1: Derivation of stresses

To determine the second Piola–Kirchhoff stress tensor, we start with the isothermal Clausius–Duhem inequality as given in Equation (7), i.e.

$$\begin{aligned}\mathbf {S} : \frac{1}{2}\dot{\mathbf {C}} - \left( \frac{\partial \psi _{\rm e}}{\partial \mathbf {C}_{\rm e}} : \dot{\mathbf {C}}_{\rm e} + \frac{\partial \psi _{\rm g}}{\partial \mathbf {B}_{\rm g}}:\dot{\mathbf {B}}_{\rm g}\right) + \mathcal {S}_0 \ge 0. \end{aligned}$$

When inserting the identities given in Eqs. (8) and (9) into the equation above, one can find

$$\begin{aligned}\mathbf {S} : \frac{1}{2}\dot{\mathbf {C}}- \frac{\partial \psi _{\rm e}}{\partial \mathbf {C}_{\rm e}} : \mathbf {F}_{\rm g}^{-T}\dot{\mathbf {C}}\mathbf {F}_{\rm g}^{-1} + \frac{\partial \psi _{\rm e}}{\partial \mathbf {C}_{\rm e}} : \left( \mathbf {L}_{\rm g}^{T}\mathbf {C}_{\rm e} + \mathbf {C}_{\rm e}\mathbf {L}_{\rm g}\right) - \frac{\partial \psi _{\rm g}}{\partial \mathbf {B}_{\rm g}} : \left( \mathbf {L}_{\rm g}\mathbf {B}_{\rm g} + \mathbf {B}_{\rm g}\mathbf {L}_{\rm g}^{T}\right) + \mathcal {S}_0 \ge 0 \end{aligned}$$

By reformulating the second term in this equation such that

$$\begin{aligned} {\frac{\partial \psi _{\rm e}}{\partial \mathbf {C}_{\rm e}} : \mathbf {F}_{\rm g}^{-T}\dot{\mathbf {C}}\mathbf {F}_{\rm g}^{-1} = 2\mathbf {F}_{\rm g}^{-1}\frac{\partial \psi _{\rm e}}{\partial \mathbf {C}_{\rm e}}\mathbf {F}_{\rm g}^{-T} : \frac{1}{2}\dot{\mathbf {C}} } \end{aligned}$$

the Clausius–Duhem inequality becomes

$$\begin{aligned}&\left( \mathbf {S} - 2\mathbf {F}_{\rm g}^{-1}\frac{\partial \psi _{\rm e}}{\partial \mathbf {C}_{\rm e}}\mathbf {F}_{\rm g}^{-T}\right) : \frac{1}{2}\dot{\mathbf {C}} + \frac{\partial \psi _{\rm e}}{\partial \mathbf {C}_{\rm e}} : \left( \mathbf {L}_{\rm g}^{T}\mathbf {C}_{\rm e} + \mathbf {C}_{\rm e}\mathbf {L}_{\rm g}\right) - \frac{\partial \psi _{\rm g}}{\partial \mathbf {B}_{\rm g}} : \left( \mathbf {L}_{\rm g}\mathbf {B}_{\rm g} + \mathbf {B}_{\rm g}\mathbf {L}_{\rm g}^{T}\right) + \mathcal {S}_0 \ge 0. \end{aligned}$$

Following the standard argumentation of Coleman and Noll (1963) and assuming that the stress response shall be independent of the rate of ${\mathbf {C}}$, we find the second Piola–Kirchhoff stress tensor to be defined as

$$\begin{aligned} { \mathbf {S} = 2\mathbf {F}_{\rm g}^{-1}\frac{\partial \psi _{\rm e}}{\partial \mathbf {C}_{\rm e}}\mathbf {F}_{\rm g}^{-T}. } \end{aligned}$$

### Appendix 1.2: Invariants of Mandel stress tensor and Kirchhoff stress tensor

The Kirchhoff stress tensor is defined as

$$\begin{aligned} \varvec{\tau } = \mathbf {F}\mathbf {S}\mathbf {F}^{T}. \end{aligned}$$

Making use of the identity $\mathbf {C}_{\rm e}\mathbf {F}_{\rm g} = \mathbf {F}_{\rm g}^{-T}\mathbf {C}$ and using a push forward operation on the second Piola–Kirchhoff stress tensor $\mathbf {S}$, the definition of the Mandel stress tensor can be rewritten as

$$\begin{aligned} \begin{aligned} \mathbf {M}&= \mathbf {C}_{\rm e} \mathbf {F}_{\rm g}\mathbf {S}\mathbf {F}_{\rm g}^{T}\\&= \mathbf {F}_{\rm g}^{-T}\mathbf {C}\mathbf {S}\mathbf {F}_{\rm g}^{T}. \end{aligned} \end{aligned}$$

With this at hand, it is easy to show that the main invariants $J_\alpha$ with $\alpha \in {1,2,3}$ are identical for both, the Mandel and the Kirchhoff stress tensor, i.e.

$$\begin{aligned} \begin{aligned} J_\alpha&= {\text {tr}}\left( \mathbf {M}^\alpha \right) \\&= {\text {tr}}\left( \left( \mathbf {F}_{\rm g}^{-T}\mathbf {C}\mathbf {S}\mathbf {F}_{\rm g}^{T}\right) ^\alpha \right) \\&= {\text {tr}}\left( \left( \mathbf {C}\mathbf {S}\right) ^\alpha \right) \\&= {\text {tr}}\left( \left( \mathbf {F}\mathbf {S}\mathbf {F}^{T}\right) ^\alpha \right) \\&= {\text {tr}}\left( \varvec{\tau }^\alpha \right) . \end{aligned} \end{aligned}$$

### Appendix 1.3: Isotropic growth model for comparison

The isotropic growth model used for comparison with the anisotropic growth model developed herein is based on the formulation of Lubarda and Hoger (2002). It uses the multiplicative split of the deformation gradient, i.e.

$$\begin{aligned} \mathbf {F} = \mathbf {F}_{\rm e} \mathbf {F}_{\rm g}, \end{aligned}$$

where the growth-related deformation gradient is defined as

$$\begin{aligned} \mathbf {F}_{\rm g} = \vartheta \mathbf {I}, \end{aligned}$$

with $\vartheta$ describing the growth induced stretch. The evolution equation of this particular model is defined in terms of the Mandel stress tensor $\mathbf {M}$ as well as a set of material parameters, i.e.

$$\begin{aligned} \dot{\vartheta } := k(\vartheta )\phi (\mathbf {M}). \end{aligned}$$

Here, the driving force $\phi$ is defined as

$$\begin{aligned} \phi := {\text {tr}}\mathbf {M} - M_{\rm crit}, \end{aligned}$$

where $M_{\rm crit}$ describes the desired homeostatic stress state that should be reached by the material. Furthermore, the growth velocity is described by

$$\begin{aligned} k(\vartheta ) := {\left\{ \begin{array}{ll} k^+\left( \frac{\vartheta ^+ - \vartheta }{\vartheta ^+ - 1}\right) ^{\gamma ^+} &{} \text {if } \phi > 0 \\ k^-\left( \frac{\vartheta - \vartheta ^-}{1 - \vartheta ^-}\right) ^{\gamma ^-} &{} \text {if } \phi < 0 , \end{array}\right. } \end{aligned}$$

with $k^+$, $k^-$ denote the expansion and contraction speed, respectively. To restrict the growth process, the parameters $\vartheta ^+$ and $\vartheta ^-$ are introduced as upper and lower thresholds of the growth induced stretch. Finally, two shape factors for the evolution are described by $\gamma ^+$ and $\gamma ^-$. For further information on this particular model, the reader is kindly referred to the original publication.

### Appendix 1.4: Transformation of invariants from intermediate to reference configuration

Reformulating the definition of the referential driving force, i.e.

$$\begin{aligned} \mathbf {M} - \varvec{\chi } = \mathbf {F}_{\rm g}\mathbf {\Sigma }\mathbf {F}_{\rm g}^{T}, \end{aligned}$$

directly yields the new definition for the volumetric invariant, i.e.

$$\begin{aligned} \begin{aligned} I_1&= {\text {tr}}\left( \mathbf {M} - \varvec{\chi }\right) \\&= {\text {tr}}\left( \mathbf {F}_{\rm g}\mathbf {\Sigma }\mathbf {F}_{\rm g}^{T}\right) \\&= {\text {tr}}\left( \mathbf {\Sigma }\mathbf {C}_{\rm g}\right) . \end{aligned} \end{aligned}$$

Making use of this relation, the deviatoric part of the driving force is given by

$$\begin{aligned} \begin{aligned} {\text {dev}}\left( \mathbf {M} - \varvec{\chi }\right)&= {\text {dev}}\left( \mathbf {F}_{\rm g}\mathbf {\Sigma }\mathbf {F}_{\rm g}^{T}\right) \\&= \mathbf {F}_{\rm g}\mathbf {\Sigma }\mathbf {F}_{\rm g}^{T} - \frac{1}{3}{\text {tr}}\left( \mathbf {F}_{\rm g}\mathbf {\Sigma }\mathbf {F}_{\rm g}^{T}\right) \mathbf {I}\\&= \mathbf {F}_{\rm g}\mathbf {\Sigma }\mathbf {C}_{\rm g}\mathbf {F}_{\rm g}^{-1} - \frac{1}{3}{\text {tr}}\left( \mathbf {\Sigma }\mathbf {C}_{\rm g}\right) \mathbf {I}\\&= \mathbf {F}_{\rm g}\left( \mathbf {\Sigma }\mathbf {C}_{\rm g} - \frac{1}{3}{\text {tr}}\left( \mathbf {\Sigma }\mathbf {C}_{\rm g}\right) \mathbf {I}\right) \mathbf {F}_{\rm g}^{-1}\\&= \mathbf {F}_{\rm g}{\text {dev}}\left( \mathbf {\Sigma }\mathbf {C}_{\rm g}\right) \mathbf {F}_{\rm g}^{-1}. \end{aligned} \end{aligned}$$

Utilizing the properties of the trace operator, the deviatoric invariant can be rewritten as

$$\begin{aligned} {\hbox {} J_2 = \frac{1}{2}{\text {tr}}\left( {\text {dev}}\left( \mathbf {\Sigma }\mathbf {C}_{\rm g}\right) ^2\right) . } \end{aligned}$$

### Appendix 1.5: Arruda–Boyce model

By introducing the volumetric deviatoric split of the elastic right Cauchy–Green tensor, i.e. $\mathbf {C}_{\rm e} = J_{\rm e}^{\frac{2}{3}}\bar{\mathbf {C}}_{\rm e}$, one can describe the elastic energy of the well-known hyperelastic material model of Arruda and Boyce (1993) as

$$\begin{aligned} \begin{aligned} \psi&= U(J_{\rm e}) + W(\bar{\mathbf {C}}_{\rm e})\\&= \frac{\kappa }{4}\left[ J_{\rm e}^2 - 1 - 2{\text {ln}}\left( J_{\rm e}\right) \right] + \mu \sum _{k=1}^{K}\frac{C_k}{N^{k-1}}\left[ \bar{I}_1^{k} - 3^{k}\right] . \end{aligned} \end{aligned}$$

Here, $\kappa$, $\mu$ and $N$ are material parameters and $C_k = \left[ \frac{1}{2}, \frac{1}{20}, \frac{11}{1050}, \frac{19}{7000}, \frac{519}{673750}\right]$ are coefficients resulting from an approximation of the inverse Langevin function. Furthermore, $\bar{I}_1 = {\text {tr}}\left( \bar{\mathbf {C}}_{\rm e}\right)$ describes the first invariant of the deviatoric part of $\mathbf {C}_{\rm e}$. For further information on this model, the reader is kindly referred to the comprehensive overview of Steinmann et al. (2012)

## Code availability

The custom routines will be made available on demand. The software package FEAP is a proprietary software and can therefore not be made available.

## References

[CR1] Ahrens J, Geveci B, Law C, Hansen CD, Johnson CR (2005). 36—paraview: an end-user tool for large-data visualization. Visualization Handbook.

[CR2] Ambrosi D, Ben Amar M, Cyron CJ, DeSimone A, Goriely A, Humphrey JD, Kuhl E (2019). Growth and remodelling of living tissues: perspectives, challenges and opportunities. J R Soc Interface.

[CR3] Arruda EM, Boyce MC (1993). A three-dimensional constitutive model for the large stretch behavior of rubber elastic materials. J Mech Phys Solids.

[CR4] Barfusz O, Brepols T, van der Velden T, Frischkorn J, Reese S (2021). A single gauss point continuum finite element formulation for gradient-extended damage at large deformations. Comput Methods Appl Mech Eng.

[CR5] Bertram A (1999). An alternative approach to finite plasticity based on material isomorphisms. Int J Plast.

[CR6] Braeu F, Aydin R, Cyron C (2019). Anisotropic stiffness and tensional homeostasis induce a natural anisotropy of volumetric growth and remodeling in soft biological tissues. Biomech Model Mechanobiol.

[CR7] Braeu F, Seitz A, Aydin R, Cyron C (2017). Homogenized constrained mixture models for anisotropic volumetric growth and remodeling. Biomech Model Mechanobiol.

[CR8] Coleman B, Noll W (1963). The thermodynamics of elastic materials with heat conduction and viscosity. Arch Ration Mech Anal.

[CR9] Cyron CJ, Aydin RC, Humphrey JD (2016). A homogenized constrained mixture (and mechanical analog) model for growth and remodeling of soft tissue. iomech Model Mechanobiol.

[CR10] Cyron CJ, Humphrey JD (2017). Growth and remodeling of load-bearing biological softtissues. Meccanica.

[CR11] de Souza Neto E, Peric D, Owen D (2008). Computational methods for plasticity: theory and applications.

[CR12] Dettmer W, Reese S (2004). On the theoretical and numerical modelling of Armstrong–Frederick kinematic hardening in the finite strain regime. Comput Methods Appl Mech Eng.

[CR13] Eckart C (1948). The thermodynamics of irreversible processes. IV. The theory of elasticity and anelasticity. Phys Rev.

[CR14] Eichinger JF, Paukner D, Szafrom JM, Aydin RC, Humphrex JD, Cyron CJ (2020). Computer-controlled biaxial bioreactor for investigating cell-mediated homeostasis in tissue equivalents. J Biomech Eng.

[CR15] Fioretta E, von Boehmer L, Motta S, Lintas V, Hoerstrup S, Emmert M (2019). Cardiovascular tissue engineering: from basic science to clinical application. Exp Gerontol.

[CR16] Fung Y (1995) Stress, strain, growth, and remodeling of living organisms. In: Theoretical, experimental, and numerical contributions to the mechanics of fluids and solids, vol 46, pp 469–482

[CR17] Geuzaine C, Remacle J-F (2009). GMSH: a three-dimensional finite element mesh generator with built-in pre- and post-processing facilities. Int J Numer Meth Eng.

[CR18] Ghazanfari S, Driessen-Mol A, Strijkers G, Baaijens F, Bouten C (2015). The evolution of collagen fiber orientation in engineered cardiovascular tissues visualized by diffusion tensor imaging. PLoS ONE.

[CR19] Göktepe S, Abilez O, Kuhl E (2010). A generic approach towards finite growth with examples of athlete's heart, cardiac dilation, and cardiac wall thickening. J Mech Phys Solids.

[CR20] Goriely A (2017). The mathematics and mechanics of biological growth, volume 45 of interdisciplinary applied mathematics.

[CR21] Himpel G, Kuhl E, Menzel A, Steinmann P (2005). Computational modelling of isotropic multiplicative growth. Comput Model Eng Sci.

[CR22] Humphrey JD, Rajagopal KR (2002). A constrained mixture model for growth and remodeling of soft tissues. Math Models Methods Appl Sci.

[CR23] Korelc J (2002). Multi-language and multi-environment generation of nonlinear finite element codes. Eng Comput.

[CR24] Korelc J (2009). Automation of primal and sensitivity analysis of transient coupled problems. Comput Mech.

[CR25] Korelc J, Stupkiewicz S (2014). Closed-form matrix exponentials and its application to finite-strain plasticity. Numer Methods Eng.

[CR26] Kröner E (1959). Allgemeine kontinuumstheorie der versetzungen und eigenspannungen. Arch. Rational Mech. Anal..

[CR27] Kuhl E, Steinmann P (2003). Mass- and volume-specific views on thermodynamics for open systems. Proc R Soc A.

[CR28] Lee EH (1969). Elastic–plastic deformation at finite strains. J Appl Mech.

[CR29] Lubarda V, Hoger A (2002). On the mechanics of solids with a growing mass. Int J Solids Struct.

[CR30] Menzel A (2005). Modelling of anisotropic growth in biological tissues. A new approach and computational aspects. Biomech Model Mechanobiol.

[CR31] Noll W (1958). A mathematical theory of the mechanical behavior of continuous media. Arch Rational Mech Anal.

[CR32] Perzyna P (1966). Fundamental problems in viscoplasticity. Adv Appl Mech.

[CR33] Perzyna P (1971). Thermodynamic theory of viscoplysticity. Adv Appl Mech.

[CR34] Reese S (2005). On a physically stabilized one point finite element formulation for three-dimensional finite elasto-plasticity. Int J Numer Meth Eng.

[CR35] Reese S, Brepols T, Fassin M, Poggenpohl L, Wulfinghoff S (2021). Using structural tensors for inelastic material modeling in the finite strain regime—a novel approach to anisotropic damage. J Mech Phys Solids.

[CR36] Reese S, Govindjee S (1998). A theory of finite viscoelasticity and numerical aspects. Int J Solids Struct.

[CR37] Rodriguez E, Hoger A, McCulloch A (1994). Stress-dependent finite growth in soft elastic tissues. J Biomech.

[CR38] Schwarze M, Reese S (2011). A reduced integration solid-shell finite element based on the EAS and the ANS concept-large deformation problems. Int J Numer Meth Eng.

[CR39] Simo J (1992). Algorithms for static and dynamic multiplicative plasticity that preserve the classical return mapping schemes of the infinitesimal theory. Comput Methods Appl Mech Eng.

[CR40] Simo J, Hughes T (1998). Computational inelasticity, volume 7 of interdisciplinary applied mathematics.

[CR41] Skalak R (1981) Growth as a finite displacement field. In: Carlson DE, Shield RT (eds) Proceedings of the IUTAM symposium on finite elasticity. Springer, Dodrecht, pp 347–355

[CR42] Skalak R, Dasgupta G, Moss M, Otten E, Dullemeijer P, Vilmann H (1982). Analytical description of growth. J Theor Biol.

[CR43] Soleimani M, Muthyala N, Marino M, Wriggers P (2020). A novel stress-induced anisotropic growth model driven by nutrient diffusion: theory, fem implementation and applications in bio-mechanical problems. J Mech Phys Solids.

[CR44] Stassi-D’Alia F (1967). Flow and fracture of materials according to a new limiting condition of yielding. Meccanica.

[CR45] Steinmann P, Hossain M, Possart G (2012). Hyperelastic models for rubber-like materials: consistent tangent operators and suitability for treloar's data. Arch Appl Mech.

[CR46] Svendsen B (2001). On the modelling of anisotropic elastic and inelastic material behaviour at large deformation. Int J Solids Struct.

[CR47] Tange O (2011). Gnu parallel: the command-line power tool. Login.

[CR48] Taylor R, Govindjee S (2020) FEAP—a finite element analysis program, University of California at Berkeley. http://projects.ce.berkeley.edu/feap/manual_86.pdf. Accessed 19 Jan 2021

[CR49] Tschoegl N (1971). Failure surfaces in principal stress space. J Polym Sci Part C Polym Symposia.

[CR50] Vladimirov I, Pietryga M, Reese S (2008). On the modelling of non-linear kinematic hardening at finite strains with application to springback—comparison of time integration algorithms. Numer Methods Eng.

[CR51] Weber G, Anand L (1990). Finite deformation constitutive equations and a time integration procedure for isotropic, hyperelastic–viscoplastic solids. Comput Methods Appl Mech Eng.

[CR52] Zahn A, Balzani D (2017). Modeling of anisotropic growth and residual stresses in arterial walls. Acta Polytech.

